# Tearing down the walls: FDA approves next generation sequencing (NGS) assays for actionable cancer genomic aberrations

**DOI:** 10.1186/s13046-018-0702-x

**Published:** 2018-03-05

**Authors:** Matteo Allegretti, Alessandra Fabi, Simonetta Buglioni, Aline Martayan, Laura Conti, Edoardo Pescarmona, Gennaro Ciliberto, Patrizio Giacomini

**Affiliations:** 1 Oncogenomics and Epigenetics, IRCSS Regina Elena National Cancer Institute, Rome, Italy; 2 Regina Elena NGS group, IRCSS Regina Elena National Cancer Institute, Rome, Italy; 3 Medical Oncology 1, IRCSS Regina Elena National Cancer Institute, Rome, Italy; 4 Pathology, IRCSS Regina Elena National Cancer Institute, Rome, Italy; 5 Clinical Pathology, IRCSS Regina Elena National Cancer Institute, Rome, Italy; 6 Biobank, IRCSS Regina Elena National Cancer Institute, Rome, Italy; 7 Scientific Direction, IRCSS Regina Elena National Cancer Institute, Rome, Italy

**Keywords:** Next generation sequencing (NGS), Precision medicine, Genomic aberrations, Regulatory issues, Ethics, Patient advocacy, Bioinformatics, Cancer screening and prevention

## Abstract

The United States Food and Drug Administration (FDA) recently approved the clinical use of two comprehensive ‘mid-size’ Next Generation Sequencing (NGS) panels calling actionable genomic aberrations in cancer. This is the first endorsement, by a regulatory body, of a new standard of care in oncology. Herein, we argue that besides its many practice-changing implications, this approval tears down the conceptual walls dividing system biology from clinical practice, diagnosis from research, prevention from therapy, cancer genetics from cancer genomics, and computational biology from empirical therapy assignment.

## Main text

On November 15^th^ 2017, the Food and Drug Administration (FDA) approved the first comprehensive NGS diagnostic assay calling actionable genomic aberrations in cancer (https://www.fda.gov/downloads/medicaldevices/productsandmedicalprocedures/invitrodiagnostics/ucm584603.pdf). The assay leverages on a 468 genes Next Generation Sequencing (NGS) panel developed at the Memorial Sloan Kettering comprehensive Cancer Center (MSKCC) in New York. The panel, known as Integrated Mutation Profiling of Actionable Cancer Targets (MSK-IMPACT), captures mutations, deletions, Copy Number Variations (CNV) and rearrangements for which target and immune therapies are approved, are being clinically tested, or are expected to be available soon [1, 2]. Few days later, on November 30^th^, FDA approved FoundationOne CDx (F1CDx) (https://www.fda.gov/downloads/medicaldevices/productsandmedicalprocedures/invitrodiagnostics/ucm584603.pdf), a similar 324 gene assay.

MSK-IMPACT and F1CDx culminate a long and winding road that connects early-day companion assays aimed at individual genomic alterations (e.g. ERBB2 amplification in the 90s) with massively parallel cancer genome sequencing efforts in more recent years (reviewed in [3, 4]). The ‘mid-size’ 468/324 gene formats of MSK-IMPACT and F1CDx stand in the middle, effectively bringing the wide breadth and foresightedness of postgenomics to precision oncology.

These FDA approvals are historical and game-changing under many respects. The scientific literature and the media have extensively covered many cutting edge innovations brought about by these assays. MSK-IMPACT profiling, in particular, was shown to recruit up to 37% of profiled patients into carefully designed next generation trials (basket and *n* of one). The tight synergy with the exceptional responder initiative at MSKCC, and the early-phase, prototypic involvement of FDA in designing the assay have also been noted. This new FDA approval strategy is important because it acknowledges the unmet needs of precision oncology, and adapts regulatory paths accordingly, facilitating future introduction and refinement of entirely new classes of clinical-grade genomic tests and drugs (https://www.fda.gov/newsevents/newsroom/pressannouncements/ucm585347.htm). Ultimately, this will contribute to align clinical research, industry pipelines, and awareness-raising actions by patient advocacy organizations.

Moreover, we feel that specific attention should be drawn on several features of these assays that are practice-changing and epistemologically impinging.

For decades, cancer genomes have been searched for somatic aberrations, whereas white blood cell DNAs from probands have been searched for germline, inheritable cancer traits. In MSK-IMPACT, germline and somatic DNAs are tested side by side, which helps discriminating potential cancer drivers from gene variants of dubious significance and clonal hematopoiesis. In adopting this format, MSK-IMPACT also provides critical information on the genetic background of patients with ‘sporadic’ cancer [5], an information that may turn out useful in future retrospective analyses. Conversely, with the inclusion of MicroSatellite Instability (MSI) surrogates in both MSK-IMPACT and F1CDx, terms such as BRCAness and ‘somatic Lynch’ [6] will become popular. The wave of next generation companion assays that will predictably follow is going to revolutionize many preconceived ideas on the interaction between genome and environment, possibly reconciling models of tumor initiation into common epistemic layouts. This will impact on strategies to control environmental carcinogens and monitor population lifestyles. Campaigns for primary as well as secondary cancer surveillance are expected to incorporate (and build on) mid-size postgenomic data, in the near future.

**Fig. 1:**
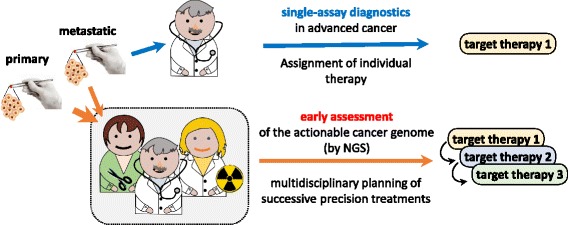
Mid-size NGS gene panels *vs* conventional molecular diagnostics. These new companion assays introduce two major conceptual changes: (a) routine molecular diagnosis no longer focuses on single genes, but encompasses a comprehensive set of alterations, inspiring (b) multidisciplinary cancer treatment at outset, and progressively narrowing down indications for single-marker companion diagnostics

MSK-IMPACT and F1CDx have deontological and ethical implications. For the first time FDA implies (some may say FDA plainly endorses the view) that each patient at an advanced cancer stage has the right to have her/his cancer genome deciphered at the highest possible level of complexity compatible with current knowledge and technology, linking molecular information to state-of-the-art systemic therapies, as they become available. For the first time extended NGS testing becomes standard of care in oncology. In the next future, NGS profiling will likely be requested at progressively earlier stages, leading to a change in the engagement rules. No longer will the oncologist request a single assay for a single therapeutic option, e.g. BRAF or ALL-RAS mutational status for specific pathway blockade in specific cancers. On the contrary, it is implicit in FDA approvals that the entire mutation catalogue will have to be made available to the medical team as soon as possible after diagnosis and much before any specific therapy becomes applicable. This will give more time to anticipate the best and the worst case for a given patient, come up with a spectrum of therapeutic options, and define a sequence of treatments aimed at optimizing response (Fig. 1). As a result, the crucial turnpike between local and systemic cancer, that usually defines the boundaries of intervention amongst surgeons, radiation therapists and medical oncologists will be blurred, multidisciplinary therapy plans being implemented early on during clinical course.

MSK-IMPACT, F1CDx and similar assays will undoubtedly affect the map of professional expertise. Bioinformaticians will be needed to empower computational system biology in the clinical practice, deciphering tumor heterogeneity, deconvoluting crucial nodes in cancer pathways, and spotting crucial cancer dependencies and vulnerabilities. A specific clinical expertise in bioinformatics is not yet canonized into higher education programs preparing these new professionals. We will also need patient manager/advisers that hinge between patients and the clinical team. As shown by MSK-IMPACT, cloud sharing is mandatory. It must be routinely applied both in the form of a de-identified public database for the wider scientific community (see for instance http://www.cbioportal.org/study?id=msk_impact_2017#summary), and as an intranet, patient-oriented resource to instruct local physicians and caregivers. With the routine application of mid-size NGS gene panels, fairly complex cancer mutational landscapes may be longitudinally captured through repeated on-treatment and post-treatment biopsies, including liquid biopsies, fueling knowledge of tumor evolution as well as screening programs of patients at risk of relapse.

Ongoing clinical trials such as the National Cancer Institute Molecular Analysis for Therapy Choice (NCI-MATCH; https://www.cancer.gov/about-cancer/treatment/clinical-trials/nci-supported/nci-match), and the American Society of Clinical Oncology Targeted Agent and Profiling Utilization Registry (ASCO TAPUR; https://www.tapur.org) will precisely measure the clinical utility of precision medicine. But whatever the future, as of today mid-size NGS gene panels raise the bar of clinical excellence worldwide, and tear down the conceptual walls dividing system biology from clinical practice, diagnosis from research, prevention from therapy, cancer genetics from cancer genomics, and computational biology from empirical therapy assignment. NGS mid-size panels are revolutionizing our operational and epistemological understanding of cancer.

## Abbreviations

CNV: Copy Number Variations
FDA: Food and Drug Administration
MSI: MicroSatellite Instability
MSK-IMPACT: Memorial Sloan Kettering Integrated Mutation Profiling of Actionable Cancer Targets.
NGS: Next Generation Sequencing
